# Detection of *Naegleria* Species in Environmental Samples from Peninsular Malaysia

**DOI:** 10.1371/journal.pone.0024327

**Published:** 2011-09-06

**Authors:** Init Ithoi, Arine Fadzlun Ahmad, Veeranoot Nissapatorn, Yee Ling Lau, Rohela Mahmud, Joon Wah Mak

**Affiliations:** 1 Department of Parasitology, Faculty of Medicine, University of Malaya, Kuala Lumpur, Malaysia; 2 School of Postgraduate Studies and Research, International Medical University, Kuala Lumpur, Malaysia; Charité-University Medicine Berlin, Germany

## Abstract

**Background:**

In Malaysia, researchers and medical practitioners are unfamiliar with *Naegleria* infections. Thus little is known about the existence of pathogenic *Naegleria fowleri*, and the resultant primary amoebic meningoencephalitis (PAM) is seldom included in the differential diagnosis of central nervous system infections. This study was conducted to detect the presence of *Naegleria* species in various environmental samples.

**Methods/Findings:**

A total of 41 *Naegleria*-like isolates were isolated from water and dust samples. All these isolates were subjected to PCR using two primer sets designed from the ITS1-ITS2 regions. The *N. fowleri* species-specific primer set failed to produce the expected amplicon. The *Naegleria* genus-specific primers produced amplicons of 408 bp (35), 450 bp (2), 457 bp (2) or 381 bp (2) from all 41 isolates isolated from aquatic (33) and dust (8) samples. Analysis of the sequences from 10 representative isolates revealed that amplicons with fragments 408, 450 and 457 bp showed homology with non-pathogenic *Naegleria* species, and 381 bp showed homology with *Vahlkampfia* species. These results concurred with the morphological observation that all 39 isolates which exhibited flagella were *Naegleria*, while 2 isolates (AC7, JN034055 and AC8, JN034056) that did not exhibit flagella were *Vahlkampfia* species.

**Conclusion:**

To date, pathogenic species of *N. fowleri* have not been isolated from Malaysia. All 39 isolates that produced amplicons (408, 450 and 457 bp) from the genus-specific primers were identified as being similar to nonpathogenic *Naegleria*. Amplicon 408 bp from 5 representative isolates showed 100% and 99.7% identity to *Naegleria philippinensis* isolate RJTM (AM167890) and is thus believed to be the most common species in our environment. Amplicons 450 bp and 457 bp were respectively believed to be from 2 new species of *Naegleria*, since representative isolates showed lower homology and had a longer base pair length when compared to the reference species in the Genbank, *Naegleria schusteri* (AJ566626) and *Naegleria laresi* (AJ566630), respectively.

## Introduction

*Naegleria* is a free-living amoeba that is ubiquitously distributed in the environment worldwide. Many species of *Naegleria* are recognized based on their small subunit ribosomal deoxyribonucleic acid (SSU rDNA), large subunit ribosomal DNA (LSU rDNA) and the internal transcribed spacer (ITS) regions, including the 5.8S rDNA [1]. However, only *Naegleria fowleri* has been shown to cause human disease, that result in primary amoebic meningoencephalitis (PAM), a rapid fatal infection of the central nervous system (CNS) [2]. PAM was most frequently reported in healthy young persons with a recent history of aquatic activities [3], [4], [5]. PAM was associated with pipe water and man-made storage ponds used by Muslims for routine ritual ablution, which involves sniffing water into the nostrils [6], [7]. *N. fowleri* infects the CNS through inhalation through the nasal passages of the trophozoite, flagellate and cyst forms that contaminate water or dust. The flagellates and cysts after transformation to trophozoites then migrate to the olfactory nerves and invade the brain causing hemorrhagic necrosis and edema [8].

In Malaysia, *Naegleria* is unfamiliar to most physicians, pathologists and laboratory workers. Up to 2010, clinicians have seldom considerd *N. fowleri* in the differential diagnosis of CNS infections. Thus the question arises as to whether PAM is underdiagnosed or that pathogenic *N. fowleri* may be rare in the environment.

*N. fowleri* was reported to cause many PAM fatal cases and was easily isolated from aquatic environments of many provinces of our neighbor Thailand [9]. As both Malaysia and Thailand have similar environmental conditions, it is likely that *N. fowleri* is also present here. Other than aquatic activities, ritual ablutions using tap water by Malaysian Muslims would potentially expose them to infection if pathogenic *N. fowleri* is present in the environment. Therefore, the present study aimed to detect *Naegleria* species in the Malaysian environment by microscopy and PCR using esthablished primer sets designed from the ITS1-ITS2 regions.

## Materials and Methods

### Collection and cultivation of samples

All sampling sites and environmental samples (dust, debris and water) were selected based on the benefit, convenience and usefulness to the public health. They were swimming pools, recreational lakes, recreational streams, mosques and air-conditioners that located at Selangor and Federal Territory, representing the most developed region of Malaysia (Table 1). There was no permits were required to collect dust, debris and water samples from all of these public sampling sites. In order to collect these environmental samples, the representatives from our research group had shown the letter entitled ‘request for permission to collect samples’ from the Head of Department of Parasitology, University of Malaya, to the authorities or the security personnels concerned, following with the explanation of the study objectives.

**Table 1:** Locations of the sampled sites at Selangor (S) and Federal Territory (FT), West Malaysia.

Designation	Location of the sampled sites	Longitude	Latitude
	**Swimming Pools**		
SP1	University Tower, Petaling Jaya (PJ), S	101.65°E	3.11°N
SP2	Pantai Panorama II, Kuala Lumpur (KL), FT	101.61°E	3.11°N
SP3	Sri Langit, Seputeh, KL, FT	101.69°E	3.12°N
SP4	Sri Seputeh, Seputeh, KL, FT	101.69°E	3.12°N
SP5	CASA Damansara, PJ, S	101.63°E	3.13°N
SP6	Prima 16, Section 17, PJ, S	101.64°E	3.13°N
SP7	Li Villas, Section 17, PJ, S	101.64°E	3.13°N
SP8	Taman Indah Persona, Section 17, PJ, S	101.65°E	3.08°N
SP9	Sport Centre University Malaya, KL, FT	101.65°E	3.12°N
SP10	Vista Angkasa, KL, FT	101.66°E	3.11°N
SP11	Pantai Hill Park, KL, FT	101.67°E	3.11°N
SP12	Pantai Panorama I, KL, FT	101.61°E	3.11°N
SP13	Vista Serdang, Serdang, S	101.69°E	3.04°N
SP14	Ehsan Ria Condominium, PJ, S	101.65°E	3.11°N
	**Recreational Lakes**		
L1	Tasik University Malaya, KL, FT	101.66°E	3.12°N
L2	Tasik Putra Jaya, KL, FT	101.71°E	3.08°N
L3	Tasik Titi Wangsa, KL, FT	101.70°E	3.18°N
L4	Tasik Perdana, KL, FT	101.68°E	3.15°N
L5	Tasik Taman Jaya, PJ, FT	101.65°E	3.11°N
L6	Tasik Taman Kelana Jaya, Kelana Jaya, S	101.62°E	3.00°N
L7	Tasik Taman Subang Jaya, S	101.60°E	3.08°N
L8	Tasik Taman Tun Dr. Ismail, PJ, S	101.63°E	3.15°N
L9	Taman Tasik Shah Alam, S	101.52°E	3.07°N
L10	Taman Tasik Permaisuri, Cheras, S	101.72°E	3.10°N
	**Recreational Streams**		
S1	Sungai Congkak, Hulu Langat, S	101.86°E	3.23°N
S2	Sungai Tekala, Hulu Langat, S	101.87°E	3.06°N
S3	Sungai Batu, Ulu Gombak, S	101.70°E	3.23°N
S4	Sungai FRIM, Kepong, S	101.63°E	3.24°N
S5	Sungai Taman Rimba Bukit Belacan, Ampang, S	101.79°E	3.15°N
	**Water tanks at Mosques**		
M1	Masjid University Malaya, KL, FT	101.65°E	3.11°N
M2	Masjid Tun Abdul Aziz, PJ, S	101.50°E	3.11°N
M3	Masjid Ar-rahman, PJ, S	101.66°E	3.12°N
M4	Masjid Aminah, PJ, S	101.55°E	3.11°N
	**Air-conditioners in Lecture Hall**		
AC1 to AC6	Medical Faculty, University Malaya, KL, FT	101.65°E	3.12°N
AC7 to AC8	International Medical University, KL, FT	101.65°E	3.06°N

The dust at the moist areas of air-conditioners and debris at the water margins of the wall of swimming pools and water tanks were collected by scrapping with sterile cotton wool (6 samples from each sampled site) and placed onto each of 6 non-nutrient agar (NNA) plates prepared from PAS (PAGE amoebae saline) overlaid with thin layers of live *Escherichia coli*. Water samples (2000 mL in sterile Schott glass-bottle) were first filtered through a nitro-cellulose membrane (1.2 µm pore size, Millipore) and the trapped debris was carefully flushed *in situ* with 6.0 mL of sterile distilled water followed by spreading 1.0 mL evenly onto each of 6 NNA-*E. coli* plates as above. The culture plates were sealed with parafilm and each set of 2 plates were incubated at room temperature (26±2°C), 37°C and 44°C, respectively, for up to 14 days. Three sets of 2 types of negative controls were carried out, each inoculated with sterile distilled water and sterile cotton wool respectively, onto NNA-*E. coli* plates and incubated as for the test samples.

### Detection of *Naegleria*-like species

**Figure 1:**
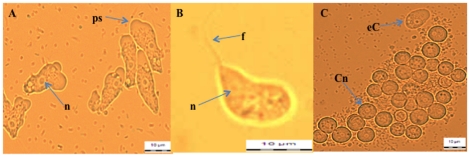
Morphological observation of *Naegleria-*like trophozoite (A, ×400), flagella (B, ×1000) and cyst (C, ×400) by using a light microscope (Olympus BX51). Nucleus (n), eruptive lobopodia / pseudopodia (ps), flagella (f), cyst with nucleus (Cn), early cyst stage (eC).

All culture plates were examined daily up to 14 days using a light microscope (Olympus BX51) before being discarded. The morphological characterictics of *Naegleria*-like trophozoites, flagellas and cysts were photographed as shown in Figure 1. Initially, culture plates were colonized with mixed organisms and positive *Naegleria*-like cells could exist as mixed isolates. Cloning of each to produce a single-species cell lines was carried out. The trophozoites were induced to flagellate by adding 10 mL of sterile distilled water and then incubated at room temperature for 60 minutes. The swimming flagellates were pipetted out and dropped into all wells of a 96-well plate. Trophozoites from isolates that failed to form the flagellate stage were gently pipetted and dropped into all the 96-well plate as described above. Wells with a single flagellate (or trophozoite) were selected, and amplified in xenic culture condition. The diameter of the cysts and the length of motile trophozoites were also determined. The cysts of the cloned cell lines were then subjected to axenisation with 3% (v/v) HCl overnight. Following the treatment, cells were seeded in protease-yeast extract-nucleic acid-folic acid-hemin (PYNFH) medium supplemented with 10% of heat inactivated fetal calf serum and 200 µg/mL gentamicin (Gibco) for cultivation. *Naegleria* isolates that proliferated well in axenic culture conditions were subsequently cultured in PYNFH medium alone.

### PCR and DNA sequencing

Two sets of primer pairs, *Naegleria fowleri* species-specific (5′- GTG AAA ACC TTT TTT CCA TTT ACA -3′ and 5′- AAA TAA AAG ATT GAC CAT TTG AAA -3′), and *Naegleria* genus-specific (5′- GAA CCT GCG TAG GGA TCA TTT -3′ and 5′- TTT CTT TTC CTC CCC TTA TTA -3′) designed from ITS regions and used previously for *Naegleria* detection [10], were used in this study. Total genomic DNA was extracted from trophozoites of *Naegleria* using the QIAamp DNA mini kit (Qiagen, Hilden, Germany). Amplification reactions were performed according to Pélandakis et al. [10]. PCR amplicons were separated by a 1.5% agarose gel prepared in 0.5×Tris-borate EDTA (TBE) buffer containing ethidium bromide. Electrophoresis was run at 100 V for 60 minutes using the 0.5×TBE buffer as running buffer. After electrophoresis, gels were viewed under UV illumination in the chamber of a BioDoc-It™ Imaging System (UVP, Cambridge, United Kingdom). Amplicon sizes were estimated by comparison with the GeneRuler 100 bp DNA ladder Plus (Fermentas Life Science, Canada).

The amplicons from 10 selected isolates were gel-purified using the QIAquick gel extraction kit (Qiagen, Hilden, Germany) prior to cloning. The purified amplicons were cloned using the InsT/Aclone ™ PCR product cloning kit (Fermentas). The DNA fragments were inserted into vectors (plasmids, pTZ57R/T) to form a DNA recombinant. The recombinant molecules were transformed into *Escherichia coli* (strain JM109, Promega)) followed by selection of the white colonies carrying recombinant plasmids with a disrupted β-galactosidase gene.

The plasmid DNA in selected recombinant colonies were confirmed by PCR amplification and were gel-purified using QIAprep® Miniprep kit (Qiagen, Hilden, Germany) prior to sequencing. The purified plasmid DNA from purified amplicons were sequenced directly at both strands using the amplification primers (forward M13/pUC (−20), 17-mer and reverse T7 promoter encoded on the pTZ57R/T vector) in an ABI PRISM™ Bigdye ™ terminator cycle sequencing ready reaction kit V.3.1 (Korea).

DNA chromatograms were examined using the Chromas software version 1.45 [11]. The forward and reverse sequences were pair-wise aligned using ClustalW software [12] and were manually refined to obtain a better consensus sequence. For nucleotide similarities search analyses, each consensus sequence was blasted against all eukaryotic nucleotide sequences archived in the GenBank database [13]. Nucleotide residues corresponding to primer regions were excluded from the similarities search analyses. The new data on DNA sequencing of all these 10 selected isolates (SP9, L1, AC1, M1, SP1, M2, S1, M4, AC7 and AC8) has been deposited in GenBank with the accession numbers (JN034047, JN034048, JN034049, JN034050, JN034051, JN034052, JN034053, JN034054, JN034055 and JN034056), respectively.

### Phylogenetic analyses

Phylogenetic analyses were based on the internal transcribed spacers (ITS) and the 5.8S sequences performed using the Molecular Evolutionary Genetics Analysis (MEGA) software [14]. The phylogenetic trees were subsequently constructed using the neighbor-joining (NJ) [15] with molecular distances estimated by the Kimura two-parameter model [16]. The bootstrap procedure was then used to evaluate the robustness of each node [17].

## Results

### Microscopic observation of *Naegleria*-like species

The trophozoite stage was normally observed after the second or third days of culture. The cyst stage could be seen after at least 5 days of cultivation. The flagellate stage was seen in the watery surface of the agar or could be induced by adding sterile distilled water to the surface agar. All the three stages are illustrated in Figure 1. Fourty-one (41) isolates from 41 locations were successfully grown in the laboratory. All these isolates showed good growth at 26±2°C and 37°C and died at 44°C. Morphologically, the ranges of cyst diameter were between 8–10 µm for all the 41 isolates. Trophozoite lengths were 15–20 µm for 26 isolates, 15–25 µm for 13 isolates, and 20–35 µm for 2 isolates. All of these isolates also showed the flagellate stage after the test for enflagellation except for 2 isolates, AC7 and AC8 (Table 2).

**Table 2:** Size and growth capability of *Naegleria*-like isolates.

Source and designation of isolate	No. of isolate	Length of trophozoite(µm)	Diameter of cyst (µm)	26±2°C	37°C	44°C	Flagellation
SP2,SP3,SP4,SP5,SP7,SP11,SP12,SP13,SP14, L2,L3,L4,L5,L6,L7,L8,L9,L10, S2,S3,S4, M3,M4, AC2,AC3,AC4	26	15–20	8–10	**+**	**+**	**−**	**+**
SP1,SP6,SP8,SP9,SP10,L1,S1,S5, M1,M2,AC1,AC5,AC6	13	15–25	8–10	**+**	**+**	**−**	**+**
AC7, AC8	2	20–35	8–10	**+**	**+**	**−**	**−**

Swimming pool (SP), recreational lake (L), recreational stream (S), water tank at mosques (M), air-conditioner (AC), present (+), absent (−).

### PCR products

**Figure 2:**
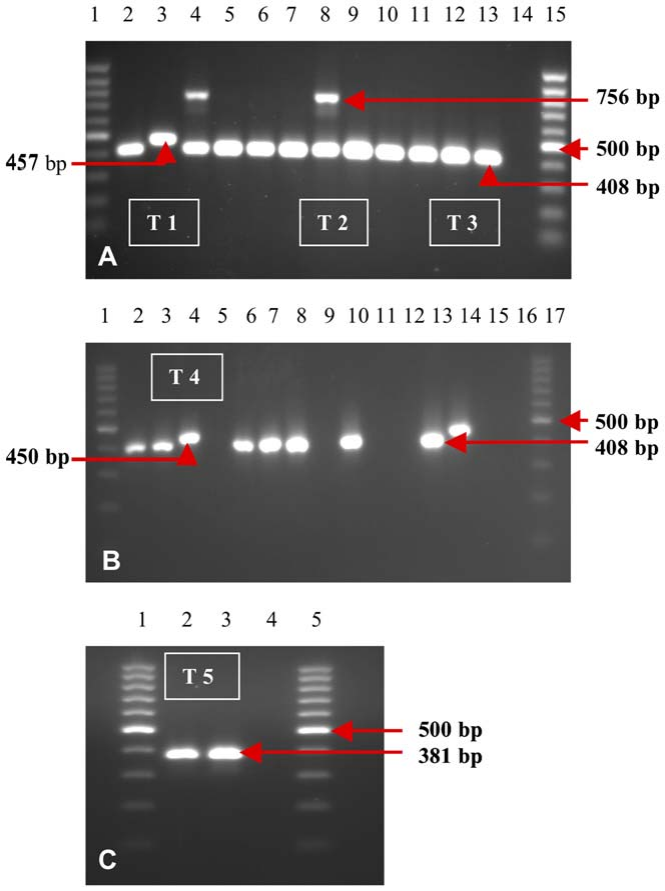
Amplicon bands revealed by genus-specific primers against *Naegleria*-like isolates. Figure 2A: 100 bp DNA ladder (lane 1&15), SP13 (lane 2), SP14 (lane 3), L1 (lane 4), L2 (lane 5), L3 (lane 6), L4 (lane 7), L5 (lane 8), L6 (lane 9), L7 (lane 10), L8 (lane 11), L9 (lane 12), L10 (lane 13), ddH_2_O (lane 14) Figure 2B: 100 bp DNA ladder (lane 1&17), S1 (lane 2), S2 (lane 3), S3 (lane 4), *Acanthamoeba-LR4 (lane 5), S4 (lane 6), S5 (lane 7), M1 (lane 8), *Acanthamoeba-Sw8 (lane 9), M2 (lane 10), *Acanthamoeba-LR10 (lane 11), *Acanthamoeba-LR11 (lane 12), M3 (lane 13), M4 (lane 14), *Acanthamoeba-L14 (lane 15), ddH_2_O (lane 16) Figure 2C: 100 bp DNA ladder (lanes1&5), AC7 (lane 2), AC8 (lane 3), ddH_2_O (lane 4).

After PCR amplification, the *N. fowleri* species-specific primer set did not show any amplicon bands against all 41 isolates. In contrast, the *Naegleria* genus-specific primer set produced amplicons against all 41 isolates, These amplicons could be differentiated into 5 groups which were labeled as T1, T2, T3, T4 and T5 represented by 457 bp, 408 and 756 bp, 408 bp, 450 bp, and 381 bp, respectively [Table 3 and Figure 2 (A, B, C)]. On the other hand, this primer set did not amplify the DNA template of *Acanthamoeba* isolates (Figure 2B).

**Table 3:** Amplicons produced by genus-specific primers against *Naegleria*-like isolates.

Source and designation of isolate	Amplicon size (bp)	Amplicontype	No. of isolate	Isolate used in DNA sequencing
SP9, SP14	457	T1	2	SP9
SP12, L1, L5	408 and 756	T2	3	L1
SP1,SP2,SP3,SP4,SP5,SP6,SP7, SP8,SP10,SP11, SP13, L2,L3, L4, L6,L7,L8, L9, L10, S2,S3,S4, S5, M1,M2,M3, AC1,AC2,AC3,AC4,AC5,AC6	408	T3	32	SP1, M1, M2, AC1
S1, M4	450	T4	2	S1, M4
AC7, AC8	381	T5	2	AC7,AC8

Swimming pool (SP), recreational lake (L), recreational stream (S), water tank at mosque (M), air-conditioner (AC).

### ITS1-5.8S-ITS2 sequence of amplicons

Analysis using the ClustaIW programme revealed that the DNA sequence of ITS1-5.8S-ITS2 of the amplicon T1 of 457 bp (from isolate SP9, JN034047) showed 98.4% homology with *Naegleria laresi* (AJ566630) with 5 base substitution (G^218^→A, C^229^→A, A^308^→G, G^328^→A and A^347^→C) and one insertion (-^364^→T) in the ITS2, resulting in 1 bp increase in length, compared to *N. laresi* (AJ566630) (Figure S1). The T2 amplicon showed two DNA fragments of 756 bp that did not give any homology to the sequences in the NCBI's database, thus only the sequence of fragment 408 bp was used in the analysis. The DNA sequence of the 408 bp amplicon of T2 (from isolate L1, JN034048) and T3 [isolates SP1 (JN034051), M1 (JN034050), M2 (JN034052), AC1 (JN034049)] showed 100% and 99.7% identity to *Naegleria philippinensis* isolate RJTM (AM167890), with no base substitution or insertions for the entire ITS1-5.8S-ITS2 region in 2 isolates [SP1 (JN034051), M2 (JN034052)] (Figure S5), and a single base substitution, T→C, in 3 isolates M1 (JN034050) (Figure S4), AC1 (JN034049) (Figure S3) and L1 (JN034048) (Figure S2) at position 120 (in 5.8S region), 252 and 227 (in ITS2 regions), repectively. Amplicon T4 (450 bp) from isolates S1 (JN034053) and M4 (JN034054), respectively, showed 95.3% and 95.0% identity to *Naegleria schusteri* (AJ566626) (Figure S6 and S7). Both isolates showed high polymorphism at the ITS2 regions with 2 base insertions (- ^281^→T, - ^353^→A) resulting in a 2 bp increase of length compared to *N. schusteri* (AJ566626). Besides that, there were 16 (C^258, 336, 361^→T, G^288^→A, G^290, 321^→C, A^302, 363^→G, T^306, 309, 346^→C, A^313^→C, T^314^→G, A^315, 319, 358^→T) and 15 (C^258, 336, 361^→T, G^288^→A, G^290, 321^→C, A^302, 363^→G, T^306, 309^→C, A^313^→C, T^314^→G, A^315, 319, 358^→T) base substitutions in the ITS2 regions of S1 (JN034053) and M4 (JN034054), respectively. Amplicon T5 (381 bp) of isolate AC8 (JN034056) showed 100% identity to *Vahlkampfia avara* (AJ698837) (Figure S9) over the entire sequence of ITS1-5.8S-ITS2, while AC7 (JN034055) (Figure S8) was 99.7% identical, with a single base substitution (T^53^→C) in the 5.8S sequence. The lengths (in bp) and differences in the ITS1, 5.8S and ITS2 rDNA sequences of representative isolates that produced amplicons T1, T2, T3, T4 and T5 and their closest relatives are summarised in Table 4.

**Table 4:** Length and position of base in the ITS1-5.8S-ITS2 sequence of the selected isolates (amplicon T1–T5) and their closest relative strain from GenBank.

Isolate (amplicon)/Reference strain	Length (position) of bp in ITS1 sequence	Length (position) of base pair in 5.8S sequence	Length (position) of base pair in ITS2 sequence	Different in sequence in comparision to reference strain
SP9 (T1) *N. laresi* AJ566630	33 (22–54)33 (1–33)	175 (55–229)175 (34–208)	162 (230–391)161 (209–369)	5 BS, 1BI
L1 (T2) *N. philippinensis* AM167890			113 (230–342)113 (209–321)	1BS
SP1 (T3) *N. philippinensis* AM167890				
MI (T3) *N. philippinensis* AM167890				
M2 (T3) *N. philippinensis* AM167890				none
AC1 (T3) *N. philippinensis* AM167890				
S1 (T4) *N. shusteri* AJ566626			155 (230–384)153 (209–361)	16BS, 2BI
M4 (T4) *N. shusteri* AJ566626				15BS, 2BI
*N. gruberi* AJ132032	33	175	113	NA
*N. australiensis* AJ132034			100	
*N. clarki* X96575			201	
*N. schusteri* AJ566626			153	
*N. gruberi* AJ132022			153	
*N. italica* X96574			162	
*N. laresi* AJ566630			161	
*N. galeacystis* X96578			181	
*N. jamiesoni* X96570	34	174	100	
*N. andersoni* X96572	35	174	100	
*N. lovaniensis* X96569	41	175	103	
*N. fowleri* AJ132018	42	175	106	
*N. fowleri* AJ132027	84	175	106	
*N. pussardi* X96571	38	174	92	
*V. ciguana* AJ973126	31	155	161	
AC7 (T5) *V. avara* AJ698837	28 (22–49)28 (1–28)	145 (50–194)145 (29–173)	162 (195–356)162 (174–335)	1 BS
AC8 (T5) *V. avara* AJ698837				none

Base substitution (BS), Base insertion (BI), not available (NA). The reference accession number is indicated at the end of the species designations that were used in the phylogenetic analysis.

**Figure 3:**
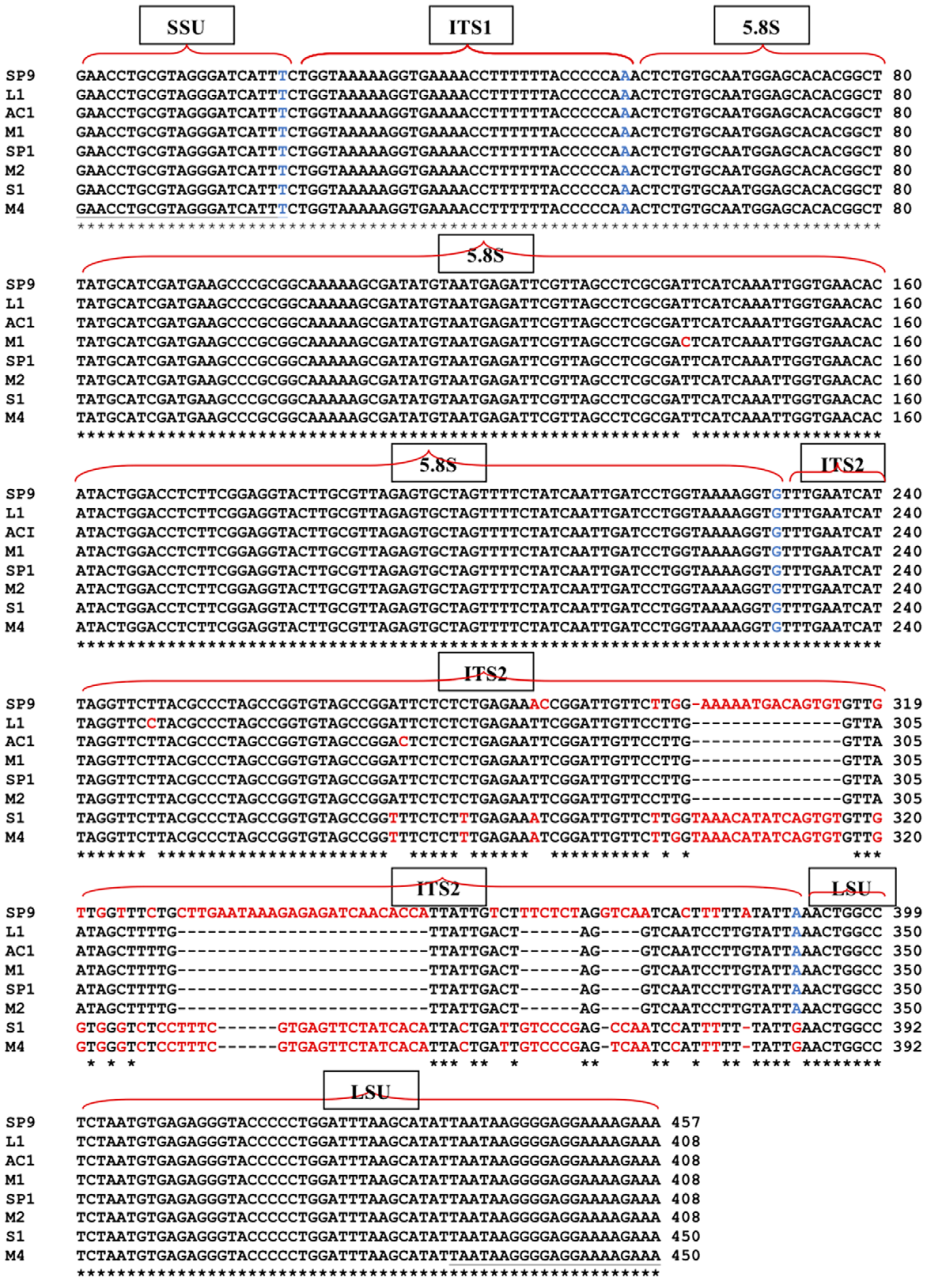
Multiple sequence alignment for amplicons T1 (SP9), T2 (L1), T3 (SP12, M1, M2, AC1) and T4 (S1, M4) using ClustalW programme. Black colour dash (gap), homologous residue (*), base substitution and insertion (red colour font), terminal base of SSU/ ITS1/ 5.8S / ITS2 or LSU sequence (blue colour font), forward and reverse primers (Underlined), small subunit ribosomal RNA (SSU), large subunit ribosomal RNA (LSU), internal transcribed spacers (ITS), 5.8S ribosomal RNA (5.8S).

Multiple sequence alignment by BLAST analyses (using ClustalW programme) against rRNA sequences (SSU-ITS1-5.8S-ITS2-LSU rDNA region) of the 8 selected isolates [SP9 (JN034047), L1 (JN034048), SP1 (JN034051), M1 (JN034050), M2 (JN034052), AC1 (JN034049), S1 (JN034053), M4 (JN034054) confirmed these are *Naegleria* species. Only isolate M1 (JN034050) showed a single base substitution (T^141^→C) at the 5.8S, wheras the other 7 isolates showed polymorphic substitutions of base sequences, deletions and insertions at their ITS2 regions (Figure 3).

### Phylogenetic analyses of representative (with amplicon T1–T4) and reference isolates

**Figure 4:**
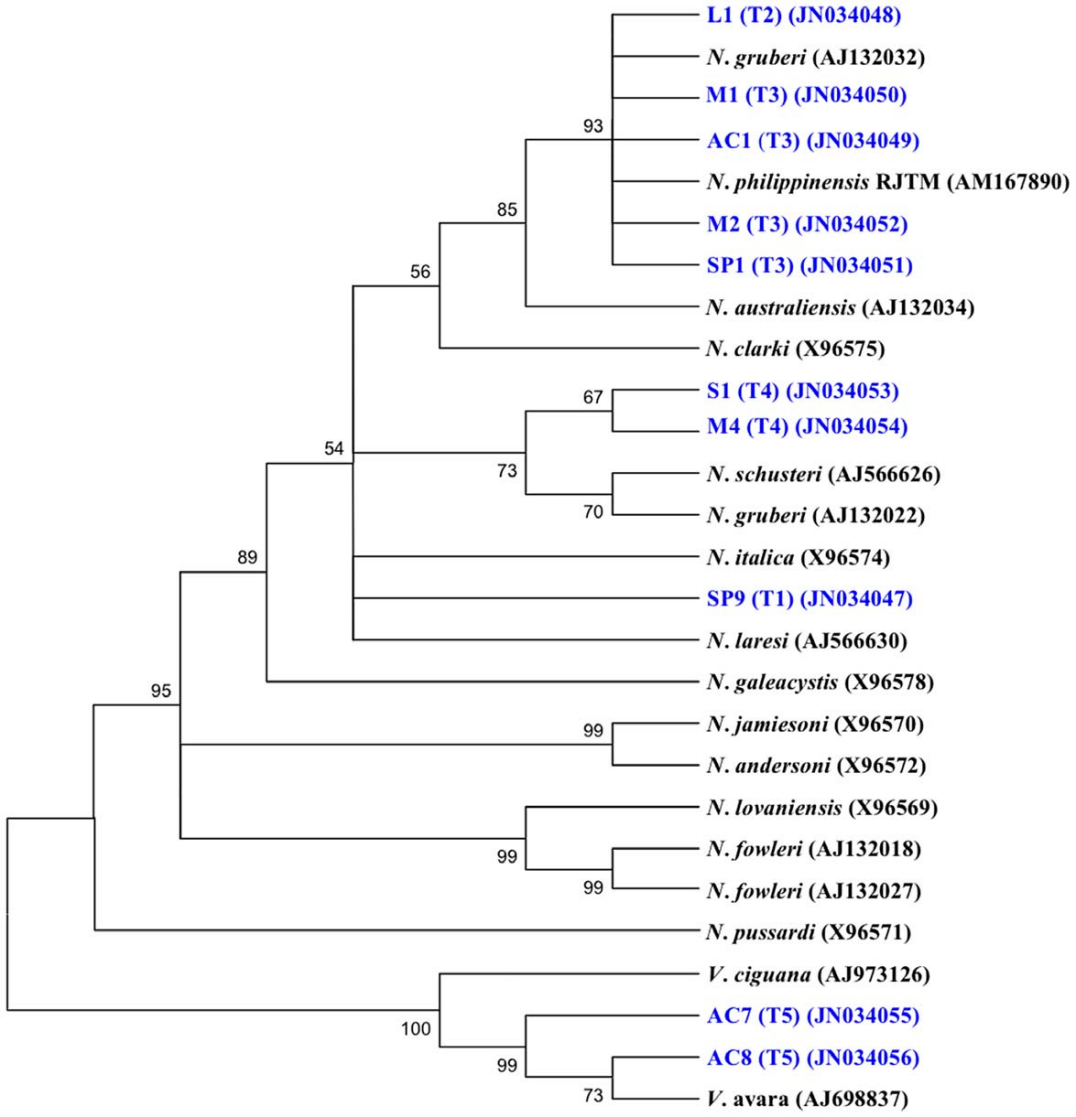
Neighbour-joining tree depicting the relationships between test isolates with amplicons (T1–T5) and reference strains of *Naegleria* and *Vahlkampfia*. Numbers at the notes are percentage-bootstrapping values on 1000 replicates, and only values of >50% are shown. GenBank accession numbers for reference sequences are indicated at the ends of the species designations.

The representative and reference isolates [L1(T2) (JN034048), *N. gruberi* (AJ132032), M1(T3) (JN034050), AC1(T3) (JN034049), *N. philippinensis* RJTM (AM167890), M2(T3) (JN034052), SP1(T3) (JN034051), *N. australiensis* (AJ132034), *N. clarki* (X96575), S1(T4) (JN034053), M4(T4) (JN034054), *N. schusteri* (AJ566626), *N. gruberi* (AJ132022), *N. italica* (X96574), SP9(T1) (JN034047), *N. laresi* (AJ566630), and *N. galeacystis* (X96578)] with similar sequences and lengths in the ITS1 (33 bp) and 5.8S (175 bp) were grouped together (Table 4, Figure 4). Two isolates, *N. jamiesoni* (X96570) and *N. andersoni* (X96572) formed a clade to each other and showed similarity to the 5.8S (174 bp). *N. fowleri* (AJ132018) and *N. fowleri* (AJ132027)) formed a clade with *N. lovaniensis* (X96569) which has an identical length of 5.8S (175 bp). *N. pussardi* (X96571) with ITS1(38 bp)-5.8S(174 bp)-ITS2(92 bp) is most distantly related within the genus *Naegleria*. Isolates AC7 (JN034055) and AC8 (JN034056) with amplicon T5, formed a clade with *Vahlkampfia avara* (AJ698837) and *Vahlkampfia ciguana* (AJ973126).

## Discussion

Initially, identification of these 41 isolates of *Naegleria*-like species was based on the morphological characteristics of the trophozoite and cyst stages. The trophozoites of all isolates showed erupted pseudophodia or lobopodia (Figure 1A) that actively moved in a unidirectional manner while the cysts had uniform smooth thick double walls. After cloning, all isolates showed a similar cyst diameter of 8–10 µm, while the motile trophozoite lengths were divided into three groups ranging from 15–20 µm (26 isolates), 15–25 µm (13 isolates) and 20–35 µm (2 isolates). All of these isolates exhibited flagella in distilled water except two from air conditioner dust samples, AC7 (JN034055) and AC8 (JN034056). The detection of flagellate (transient form trophozoite) stage was one of the important indicators for the rapid screening to differentiate the *Naegleria* from other free living amoebae especially *Acanthamoeba* species [18]. However, it was reported that some of the *Naegleria* species (eg: *Naegleria chilensis* and *Naegleria indonesiensis*) were unable to enflagellate [19], as seen with isolates AC7 (JN034055) and AC8 (JN034056) in this study. All the 41 isolates showed good growth at room temperature (26±2°C) and 37°C, but died at 44°C. Pathogenic *N. fowleri* was reported to survive up to 45°C [20] and a temperature range of 30 to 40°C was suggested to be associated with increased occurrence of *N. fowleri* in thermally elevated environments [21], [22]. In this study we used the PCR-based detection in order to confirm the *Naegleria* species that were present in our environment.

We excluded the use of pathogenic *N. fowleri* as a control because of restrictions in bringing the pathogenic organisms into the country. We used 2 primer sets, the *N. fowleri* species-specific and *Naegleria* genus-specific, designed from the ITS1-ITS2 region by Pélandakis et al. [10], which had previously provided successful results.

The ribosomal ITS sequence was reported to be a powerful tool for detecting inter- and intra-species differences of various organisms, including *Cryptosporidium parvum* [23], trichomonadid protozoa [24], *Naegleria* [25] and *Vahlkampfia* species [26]. As in *Naegleria*, the differences between genus (inter-species) and species (intra-species of *N. fowleri*) were due to the polymorphism sequence that occurred at the ITS2 and ITS1 regions, respectively [27]. The ITS region was also being used for the identification of the new *Naegleria* isolates [10], [28].

In the current study, the species-specific primer set for *N. fowleri* failed to amplify and did not produce any amplicon against all the 41 isolates, while the *Naegleria* genus-specific primer sets successfully amplified the DNA template from all isolates, producing 5 DNA amplicons consisting of 381, 408, 450, 457 and 756 bp, respectively. The amplicon of 408 bp was the most common, produced by 35 isolates, whereas 381, 450 and 457 bp amplicons were produced by 2 isolates each, namely, AC7 (JN034055) and AC8 (JN034056), S1 (JN034053) and M4 (JN034054), and SP9 (JN034047) and SP14, respectively (Table 3). Subsequently, the 756 bp fragment was belived to be from organisms that contaminated the 3 isolates (SP12, L1 (JN034048), L5), although all possible causes of contamination in the *Naegleria* culture was considered prior to DNA extraction. These 3 isolates were originally from swimming pools and recreational lakes. Production of various amplicons by this *Naegleria* genus-specific primer set have been reported previously by Pélandakis and Pernin [29], with amplicons of 800, 600 and 500 bp showing homology with *Hartmanella*, *Vahlkampfia* and *Willaertia*, respectively. Neverthelless, this *Naegleria* genus-specific primer set did not amplify the *Acanthamoebe*-like species.

Of the 41 isolates, 10 isolates produced amplicons of 408 bp [SP1 (JN034051), M1 (JN034050), M2 (JN034052), AC1 (JN034049) and L1(JN034048)], 450 bp [S1 (JN034053) and M4 (JN034054)], 457 bp [SP9 (JN034047)] and 381 bp [AC7 (JN034055) and AC8 (JN034056)], were selected for the detection of DNA sequences of the SSU-ITS1-5.8S-ITS2-LSU rDNA region of the respective amplicon. SP1 (JN034051) and SP9 (JN034047) were isolated from swimming pools frequently used by local and foreign students of the University Malaya. M1 (JN034050), M2 (JN034052), M4 (JN034054) were from water-tanks walls and the water used by thousands of Muslims to perform ritual ablutions. AC1 (JN034049) was isolated from dust at the air-conditioner in a frequently used lecture theatre at a Medical Faculty. L1 (JN034048) was isolated from water of the University's lake that actively used by the students at the evening as their water sports. S1 (JN034053) was from a favourite recreational stream (Sungai Congkak). AC7 (JN034055) and AC8 (JN034056) were from air-conditioners in the laboratories at another University in Kuala Lumpur. The 408 bp amplicon that was produced by isolates SP1 (JN034051) and M2 (JN034052) showed 100% identy in the entire lengths and sequences of ITS1(33 bp)-5.8S(175 bp)-ITS2(113 bp) against *Naegleria philippinensis* isolate RJTM (AM167890). Three isolates [M1 (JN034050), AC1 (JN034049) and L1 (JN034048) showed differences due to a single base substitution (T→C), at the position 120 (in the 5.8S), 252 and 227 (at the ITS2), respectively against *Naegleria philippinensis* isolate RJTM (AM167890). Isolates M1 (JN034050), AC1 (JN034049) and L1 (JN034048) were proposed to be placed under intra-species or strains of this *N. philippinensis* species. The sequences of both amplicon 450 bp and 457 bp from the respective isolates showed high polymorphism at the ITS2 regions, with addition of a longer length as compared to the reference species in the Genbank. Therefore, the isolates with 450 bp [S1 (JN034053) and M4 (JN034054)] and 457 bp [SP9 (JN034047)] were proposed to be included under two new species of *Naegleria*, respectively. For isolate SP9 (457 bp) (JN034047), there were 5 base substitutions and 1 base insertion, resulting in 1 bp increase in length but a 98.4% identical sequence with *Naegleria laresi* (AJ566630). S1 (JN034053) and M4 (JN034054) showed 16 and 15 base substitutions respectively, with a 2 base insertions in both isolates, resulting in 2 bp increase in length compared to *Naegleria schusteri* (AJ566626). Additionally, S1 (JN034053) and M4 (JN034054) were proposed to be strains of a new *Naegleria* species, because of their intra-species variation due to the differences of only a single base substitution (T^346^→C) in S1 (JN034053) at ITS2 region. De Jonckheere [1] reported previously that the polymorphism of ITS2 sequences could determine the species within the genus (inter-species) of *Naegleria*. All the strains from similar species (intra-species) of all *Naegleria* (except *Naegleria byersi* and *Naegleria andersoni*) showed identical lengths of the ITS2 sequences. In one strain each of *N. byersi* and *N. andersoni*, a difference in 1 bp has been detected.

For the largest amplicon of 756 bp, it was initially suspected to be homologous with contaminated *Hartmanella*, since this fragment is nearest to fragment 800 bp that was reported to be homologous with *Hartmanella* [29]. However after sequencing this amplicon from isolate L1 (JN034048), it did not show homology to any of the sequences from the GenBank. The smallest amplicon of 381 bp produced by 2 isolates [AC8 (JN034056, AC7 (JN034055)] were 100 and 99.7% identical with *Vahlkampfia avara* (AJ698837), respectively. Only isolate AC7 (JN034055) showed a difference in a single base substitution, T^53^→C) in the 5.8S sequence. This finding was different from Pélandakis and Pernin [29], who obtained a PCR product of approximately 600 bp for *Vahlkampfia* spp.

Multiple sequence alignment by BLAST analyses using the ClustalW programme against rRNA sequences (SSU-ITS1-5.8S-ITS2-LSU rDNA region) of amplicons 408 bp [L1 (JN034048), AC1 (JN034049), M1 (JN034050), SP1 (JN034051), M2 (JN034052)], 450 bp [S1 (JN034053), M4 (JN034054)] and 457 bp [SP9, (JN034047)] were confirmed as *Naegleria* species. With the exeption of M1 (JN034050), all 9 selected isolates showed polymorphism sequences (base substitutions, deletions and insertions) at the ITS2. Phylogenetic analyses using 3 different algorithms [neighbour-joining (Figure 4), minimum evolution and maximum parsimony (Figure not showed)] clearly separated the sequences of SSU-ITS1-5.8S-ITS2-LSU rDNA region of selected isolates with 408 bp, 450 bp and 457 bp (*Naegleria* spp.) from the 381 bp of the *Vahlkampfia* spp. The 408 bp sequences showed intraspecies variation that clustered together with *N. philippinensis* (AM167890). The 450 bp sequences had a relationship to *N. schusteri* (AJ566626) and *N. gruberi* (AJ132022), while the 381 bp sequences formed a clade with *V. avara* (AJ698837) and *V. ciguana* (AJ973126). Both, strains selected (from this study) and the reference isolates provided by GenBank, have similar length and sequence in the ITS1 (33 bp) – 5.8S (175 bp) regions. Polymorphism in the ITS2 sequences is an important feature used for the detection of intra- and interspecies variation of non-pathogenic *Naegleria* as in this study. This finding supported the findings of De Jonckheere [1], [30] who showed differences in the sequence of the ITS2 region in species of the genus *Naegleria*.

In this study, both results from PCR- and morphology-based detections revealed that all 39 isolates with flagella were *Naegleria*, while 2 isolates [AC7 (JN034055) and AC8 (JN034056)] with no flagella were *Vahlkampfia* species. For the *Vahlkampfia species*, besides being unable to enflagellate, its viable trophozoites were longer in length (up to 35 µm) and were pinkish in colour.

Subsequently, all the tests such as temperature tolerance, using an *N. fowleri* species-specific primer set and rDNA sequencing failed to detect *N. fowleri* from our environment. Therefore, up to this date we have not been able to isolate *N. fowleri* in the state of Selangor and the Federal Territory of Kuala Lumpur and Putra Jaya. This may explain why PAM fatal cases have not been reported from these areas. On the other hand, all three species of *Naegleria* detected by ITS1-ITS2 were present in both, chlorinated (from swimming pools and water tanks) and non-chlorinated water (from lakes and streams). Thus our chlorinated water did not affect the viability of *Naegleria* species. Therefore, *N. fowleri* contamination of chlorinated as well as non-chlorinated water, could pose a danger to public health.

Geographically, Malaysia has less aquatic environments compared to Thailand, thus explaining why Malaysia has less surface water related activities. This does not mean that our population is not exposed to *N. fowleri*. Most Malaysian Muslims frequently use water for their ritualistic washing before praying, which may espose them to the risk of amoebae entering the nostrils if domestic water is contaminated with *N. fowleri*. In addition the northen region of Peninsular Malaysia [Perlis (100.13°E longitude, 6.40°N latitude), Kedah (100.30°E longitude, 6.10°N latitude) and Kelantan (102.25°E longitude, 6.10°N latitude)] that borders with Thailand (which has reports of *N. fowleri* in their aquatic environment in several provinces), has floods due to heavy rainfall 2–3 times yearly. Most children would take the opputunity to play and engage in water-related activities during this time. Therefore, detection of *N. fowleri* should also be caried out in this region, especially during flooding. Clinicians should be be aware of PAM and include it in the differential diagnosis of meningoencephalitis. Preventive and control measures include health education for the public and awareness among medical practioners and adequate treatment of public water supplies.

### Conclusion

Temperature tolerance tests, PCR and DNA sequencing revealed that only non-pathogenic *Naegleria* species could be isolated from the central region of peninsula Malaysia. Strains of *Naegleria philippinensis* (with amplicon 408 bp product with *Naegleria* genus specific primer sets) were the most commonly isolated. The other two amplicons, 450 bp and 457 bp, were believed to be from new species, where they showed sequence polymorphism at the ITS2 regions and an ncreased length of base pairs as compared to the reference species, *Naegleria schusteri* (AJ566626) and *Naegleria laresi* (AJ566630), respectively. All these three species produced flagella and could be detected in chlorinated and non-chlorinated water as well as in dust samples. *Vahlkampfia* species (with amplicon of 381 bp) shows *Naegleria*-like morphology but do not produce flagella.

## Supporting Information

Figure S1 **Pair wise sequence alignment of** ***Naegleria laresi*** **AJ566630 (NL) and isolate SP9, JN034047 (amplicon T1) using ClustalW programme.** Homologous residue (*), non homologous residue (blank), base substitution or insertion (red colour font), terminal base of ITS1, 5.8S and ITS2 sequence (blue colour fond).
(TIF)

Figure S2 **Pair wise sequence alignment of** ***Naegleria philippinensis*** **RJTM, AM167890 (NP) and isolate L1, JN034048 (T2) using ClustalW programme.** Homologous residue (*), non homologous residue (blank), base substitution (red colour font), terminal base of ITS1and 5.8S sequence (blue colour fond).
(TIF)

Figure S3 **Pair wise sequence alignment of** ***Naegleria philippinensis*** **RJTM, AM167890 (NP) and isolate AC1, JN034049 (T3) using ClustalW programme.** Homologous residue (*), non homologous residue (blank), base substitution (red colour font), terminal base of ITS1and 5.8S sequence (blue colour fond).
(TIF)

Figure S4 **Pair wise sequence alignment of** ***Naegleria philippinensis*** **RJTM, AM167890 (NP) and isolate M1, JN034050 (T3) using ClustalW programme.** Homologous residue (*), non homologous residue (blank), base substitution (red colour font), terminal base of ITS1and 5.8S sequence (blue colour fond).
(TIF)

Figure S5 **Pair wise sequence alignment of** ***Naegleria philippinensis*** **RJTM, AM167890 (NP) and isolate SP1, JN034051 (T3) using ClustalW programme.** Homologous residue (*), terminal base of ITS1and 5.8S sequence (blue colour fond). This pair wise also revealed by clone M2, JN034052 (T3).
(TIF)

Figure S6 **Pair wise sequence alignment of** ***Naegleria schusteri*** **AJ566626 (NS) and isolate S1, JN034053 (T4) using ClustalW programme.** Homologous residue (*), non homologous residue (blank), base substitution or insertion (red colour font), terminal base of ITS1, 5.8S and ITS2 sequence (blue colour fond).
(TIF)

Figure S7 **Pair wise sequence alignment of** ***Naegleria schusteri*** **AJ566626 (NS) and isolate M4, JN034054 (T4) using ClustalW programme.** Homologous residue (*), non homologous residue (blank), base substitution or insertion (red colour font), terminal base of ITS1, 5.8S and ITS2 sequence (blue colour fond).
(TIF)

Figure S8 **Pair wise sequence alignment of** ***Vahlkampfia avara*** **AJ698837 (VA) and isolate AC7, JN034055 (T5) using ClustalW programme.** Homologous residue (*), non homologous residue (blank), base substitution or insertion (red colour font).
(TIF)

Figure S9 **Pair wise sequence alignment of** ***Vahlkampfia avara*** **AJ698837 (VA) and isolate AC8, JN034056 (T5) using ClustalW programme.** Homologous residue (*).
(TIF)
