# Supplementary figures and images for: Detection of Naegleria Species in Environmental Samples from Peninsular Malaysia
Source: PLoS One. 2011 Sep 6;6(9):e24327. doi: 10.1371/journal.pone.0024327 (PMC3167841; doi:10.1371/journal.pone.0024327)

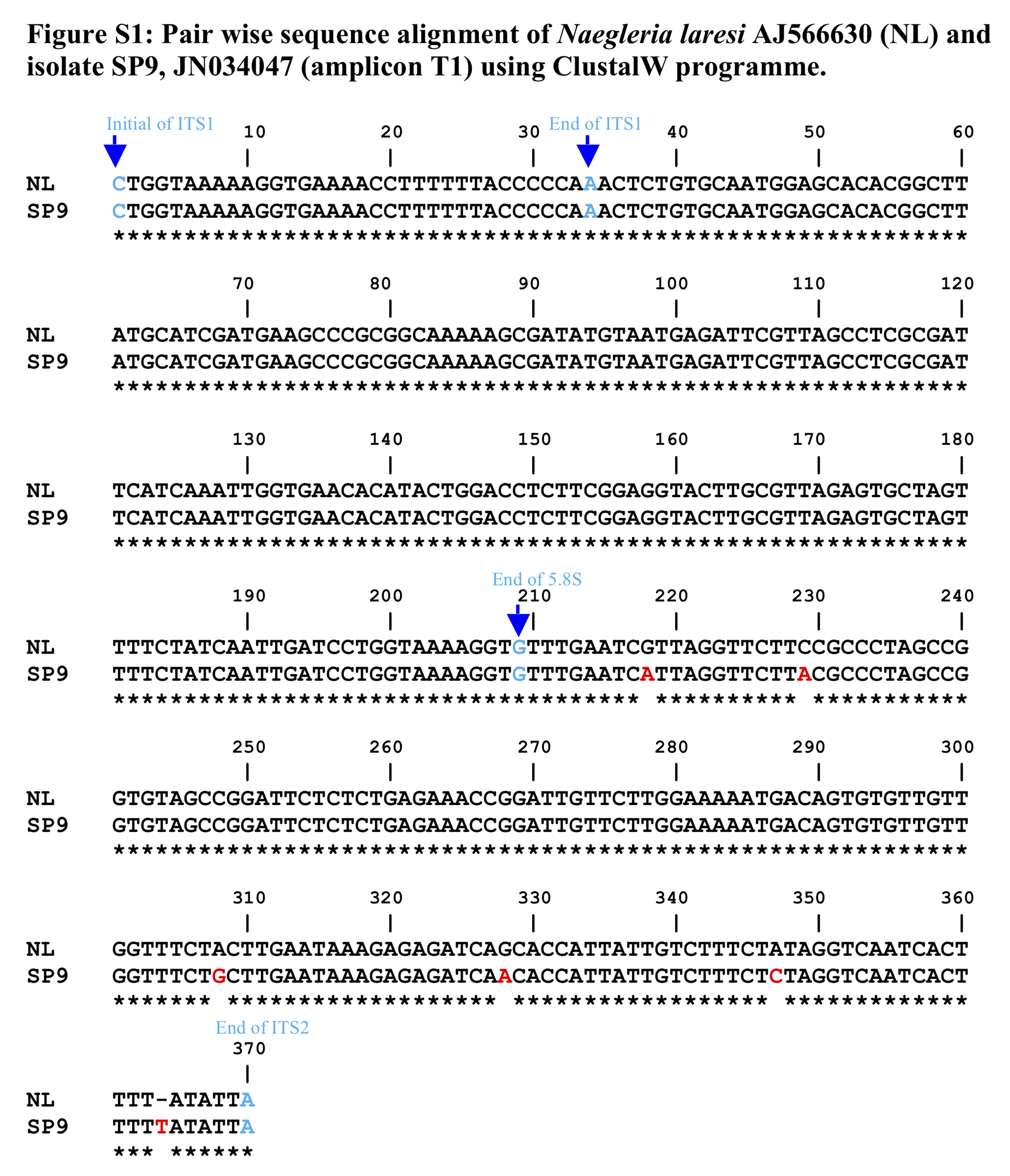

Supplement: Figure S1 — Pair wise sequence alignment of Naegleria laresi AJ566630 (NL) and isolate SP9, JN034047 (amplicon T1) using ClustalW programme. Homologous residue (*), non homologous residue (blank), base substitution or insertion (red colour font), terminal base of ITS1, 5.8S and ITS2 sequence (blue colour fond). (TIF) [file pone.0024327.s001.tif]

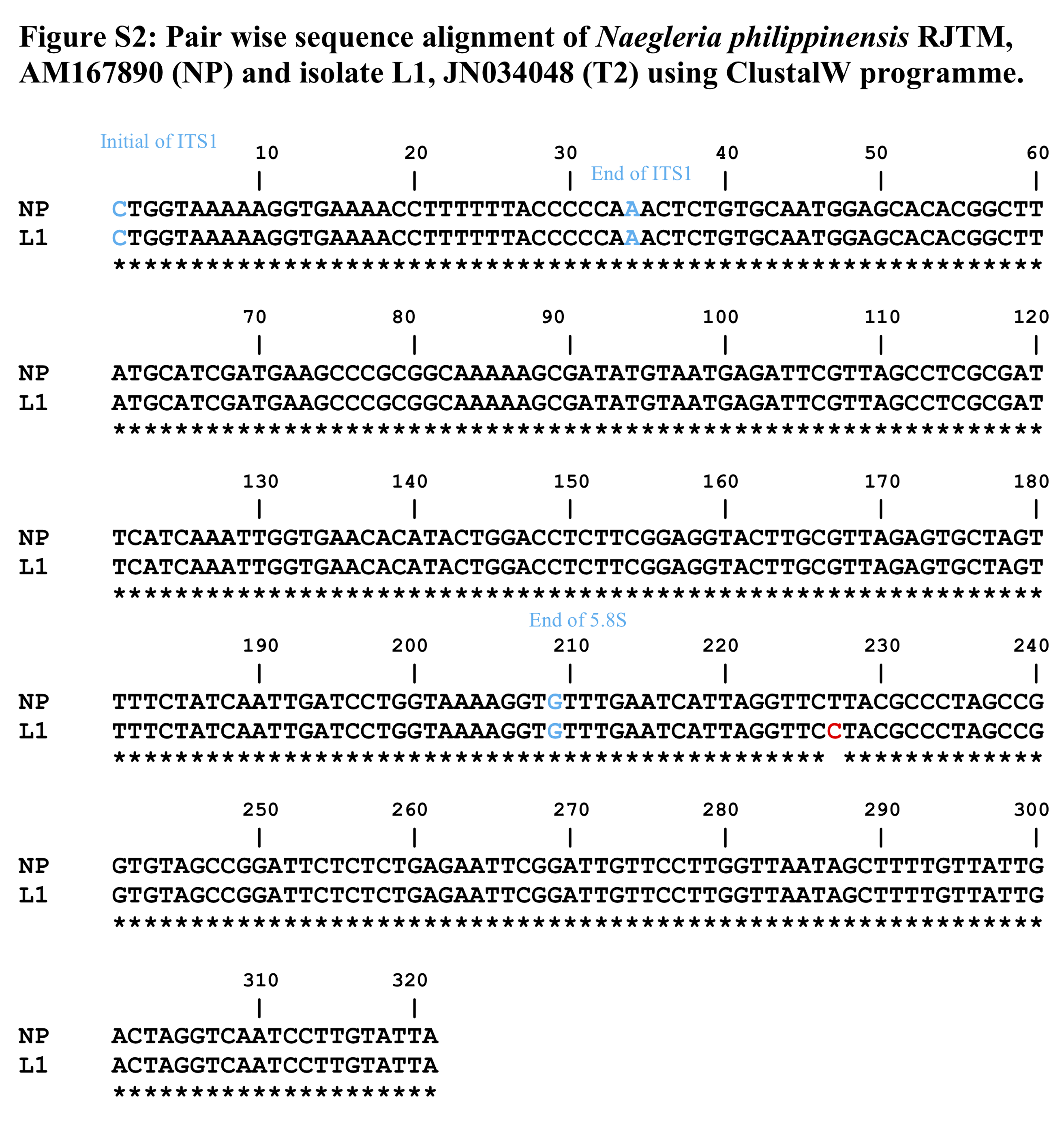

Supplement: Figure S2 — Pair wise sequence alignment of Naegleria philippinensis RJTM, AM167890 (NP) and isolate L1, JN034048 (T2) using ClustalW programme. Homologous residue (*), non homologous residue (blank), base substitution (red colour font), terminal base of ITS1and 5.8S sequence (blue colour fond). (TIF) [file pone.0024327.s002.tif]

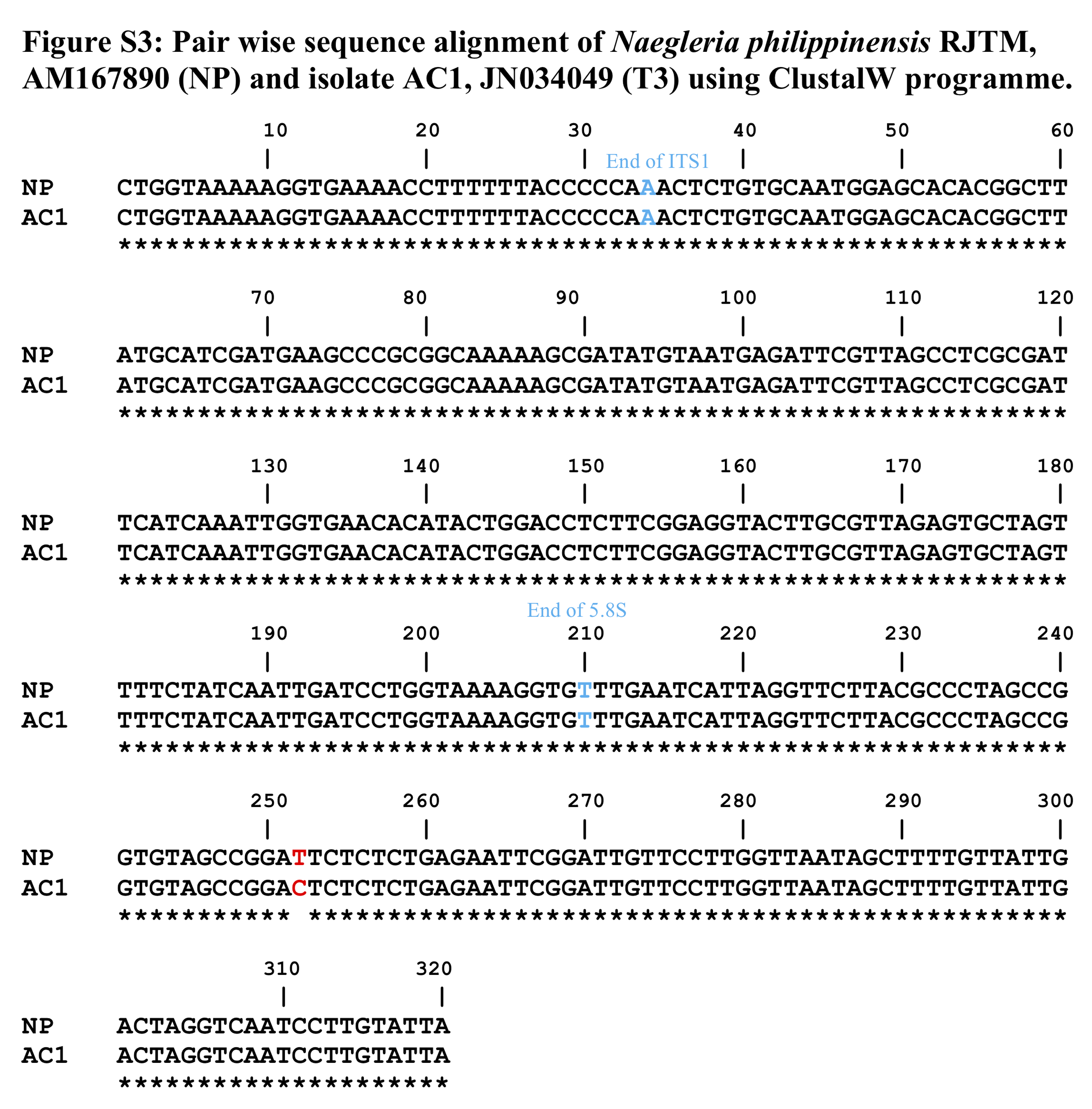

Supplement: Figure S3 — Pair wise sequence alignment of Naegleria philippinensis RJTM, AM167890 (NP) and isolate AC1, JN034049 (T3) using ClustalW programme. Homologous residue (*), non homologous residue (blank), base substitution (red colour font), terminal base of ITS1and 5.8S sequence (blue colour fond). (TIF) [file pone.0024327.s003.tif]

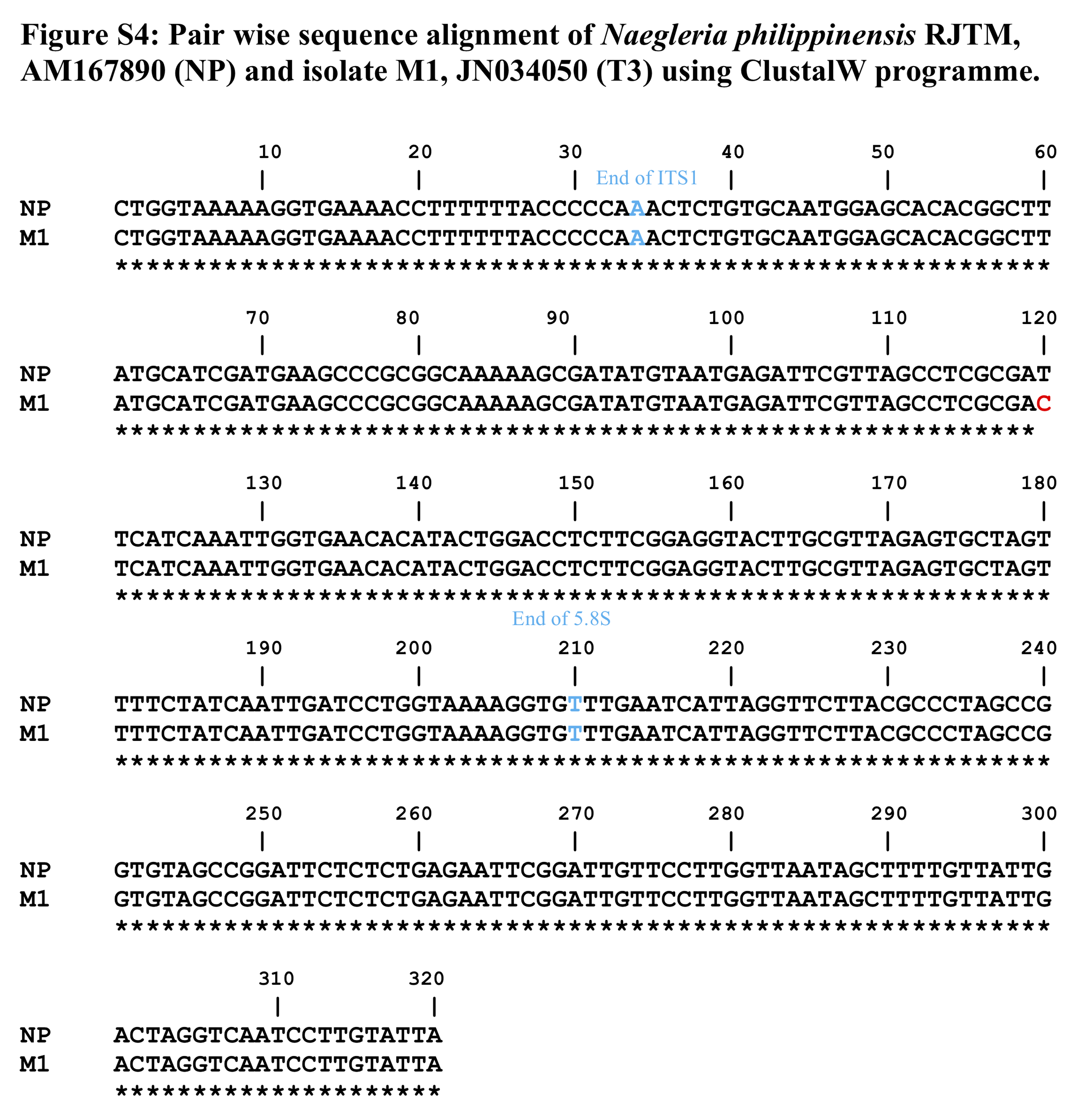

Supplement: Figure S4 — Pair wise sequence alignment of Naegleria philippinensis RJTM, AM167890 (NP) and isolate M1, JN034050 (T3) using ClustalW programme. Homologous residue (*), non homologous residue (blank), base substitution (red colour font), terminal base of ITS1and 5.8S sequence (blue colour fond). (TIF) [file pone.0024327.s004.tif]

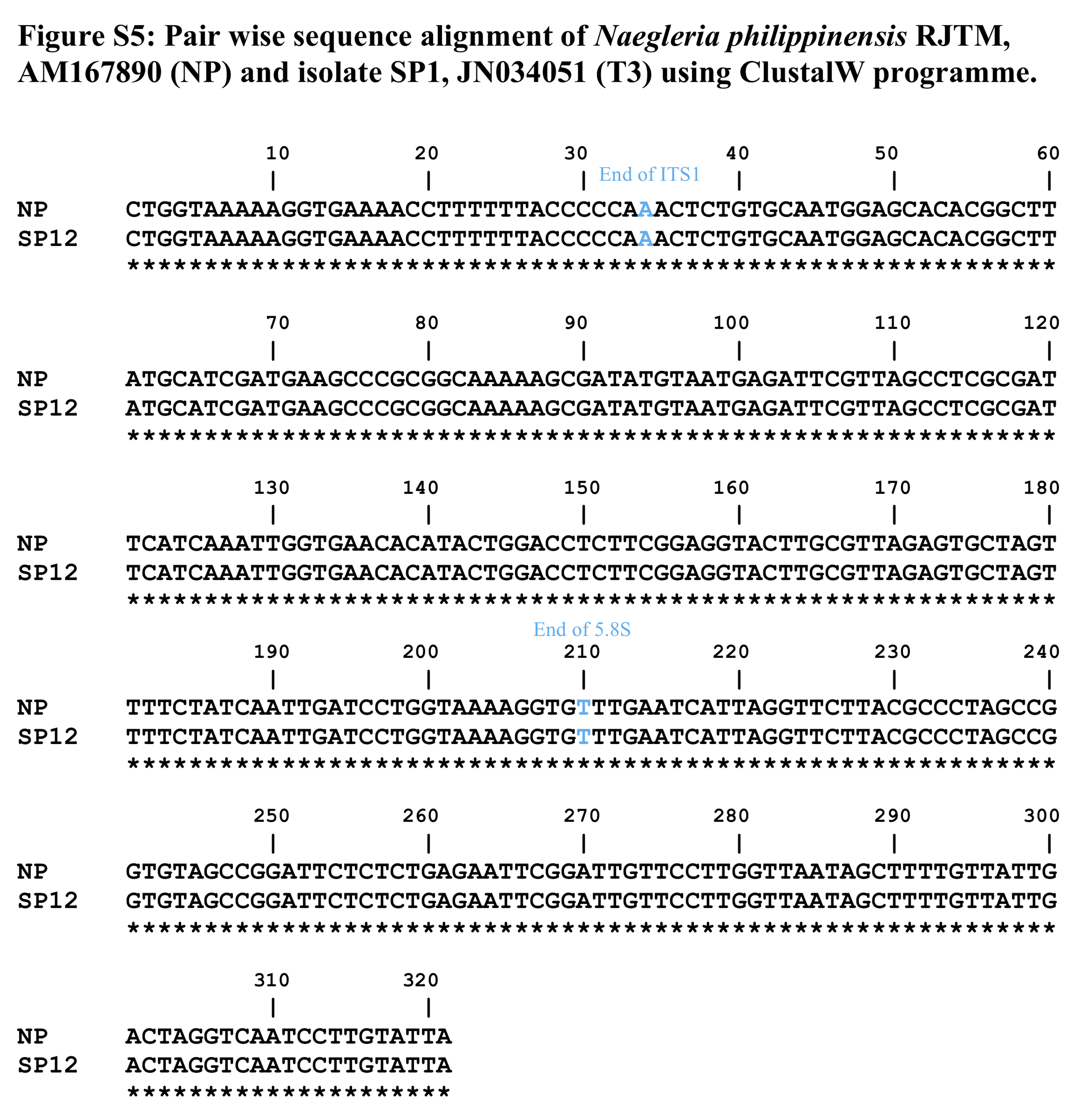

Supplement: Figure S5 — Pair wise sequence alignment of Naegleria philippinensis RJTM, AM167890 (NP) and isolate SP1, JN034051 (T3) using ClustalW programme. Homologous residue (*), terminal base of ITS1and 5.8S sequence (blue colour fond). This pair wise also revealed by clone M2, JN034052 (T3). (TIF) [file pone.0024327.s005.tif]

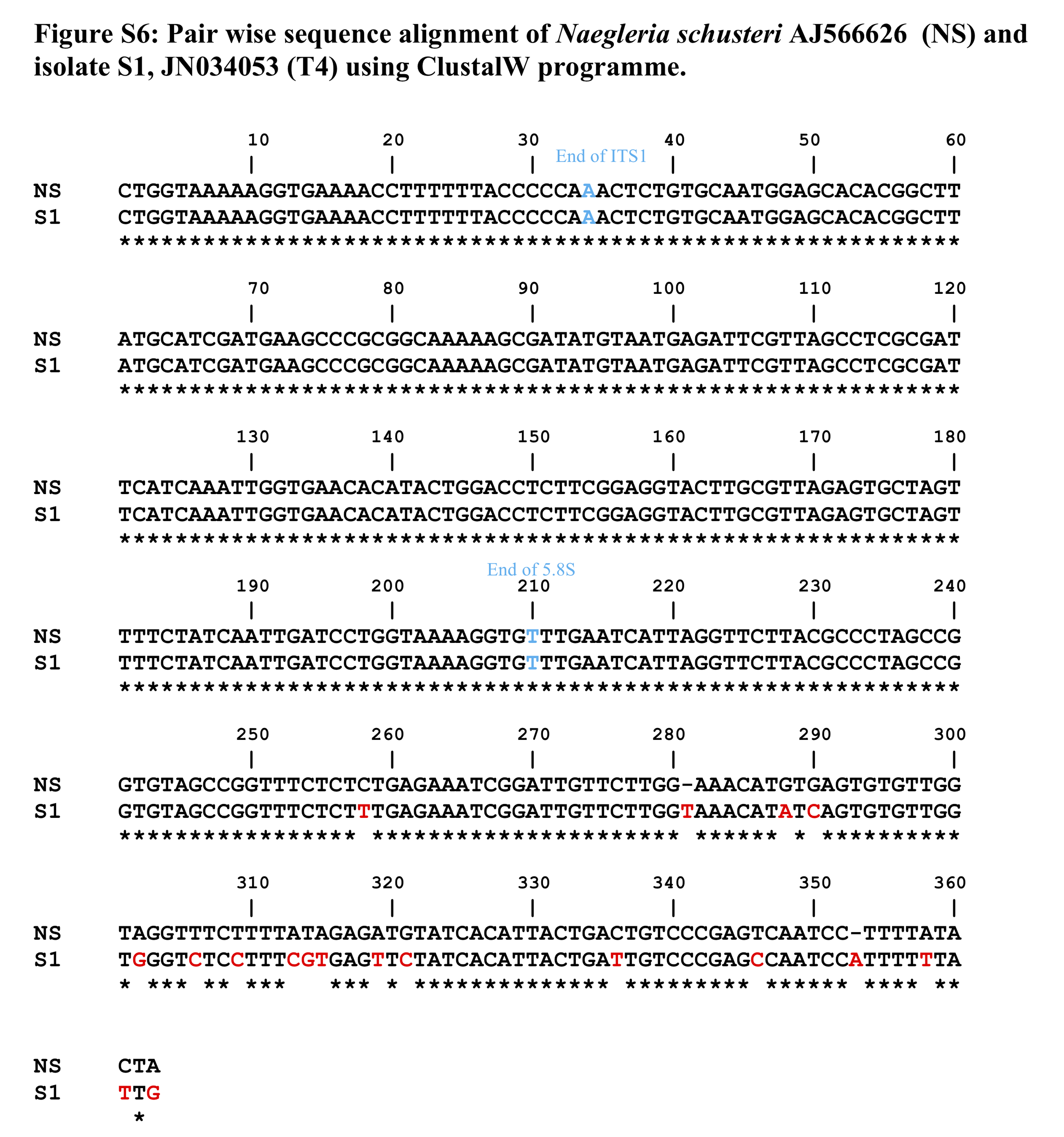

Supplement: Figure S6 — Pair wise sequence alignment of Naegleria schusteri AJ566626 (NS) and isolate S1, JN034053 (T4) using ClustalW programme. Homologous residue (*), non homologous residue (blank), base substitution or insertion (red colour font), terminal base of ITS1, 5.8S and ITS2 sequence (blue colour fond). (TIF) [file pone.0024327.s006.tif]

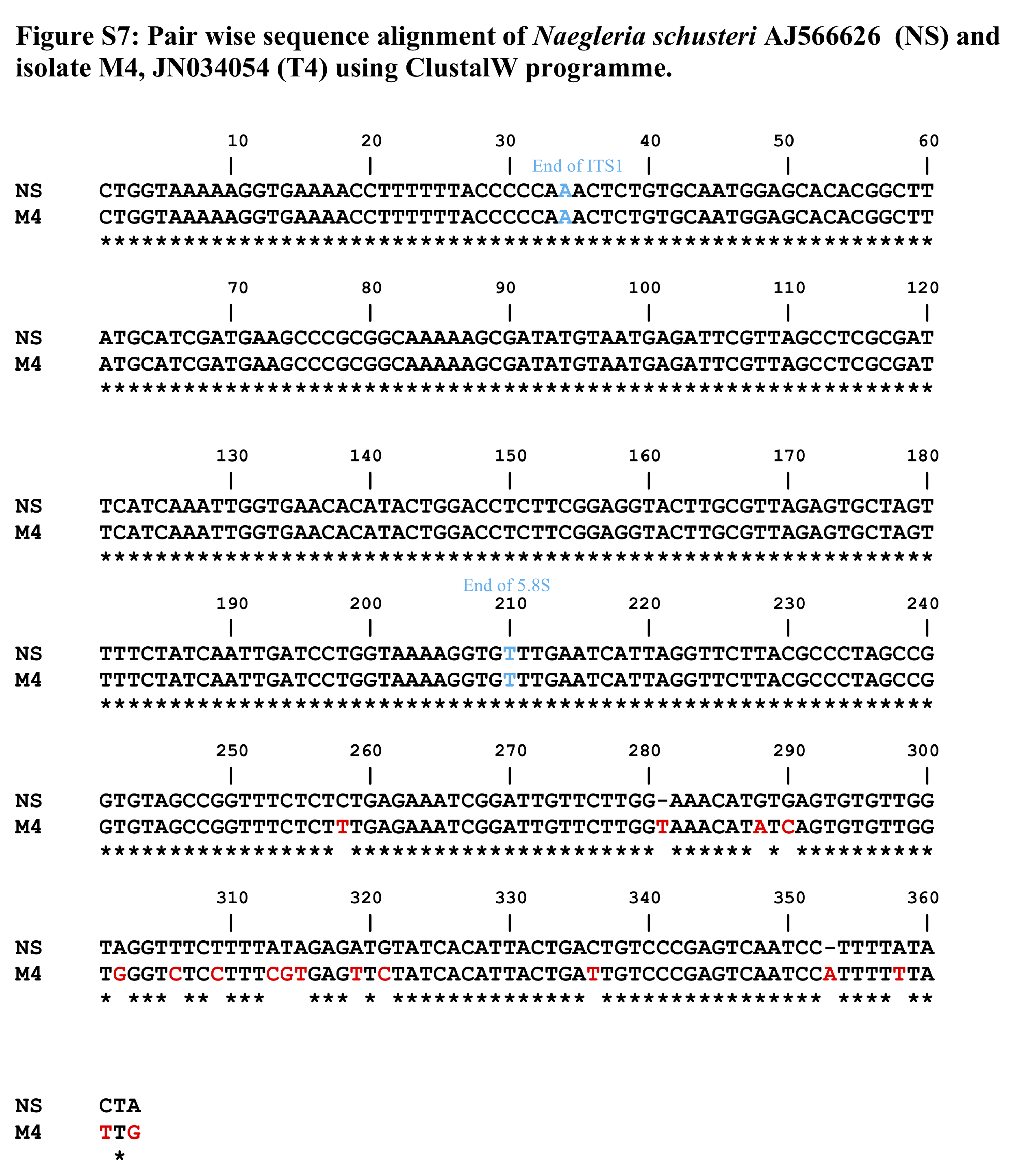

Supplement: Figure S7 — Pair wise sequence alignment of Naegleria schusteri AJ566626 (NS) and isolate M4, JN034054 (T4) using ClustalW programme. Homologous residue (*), non homologous residue (blank), base substitution or insertion (red colour font), terminal base of ITS1, 5.8S and ITS2 sequence (blue colour fond). (TIF) [file pone.0024327.s007.tif]

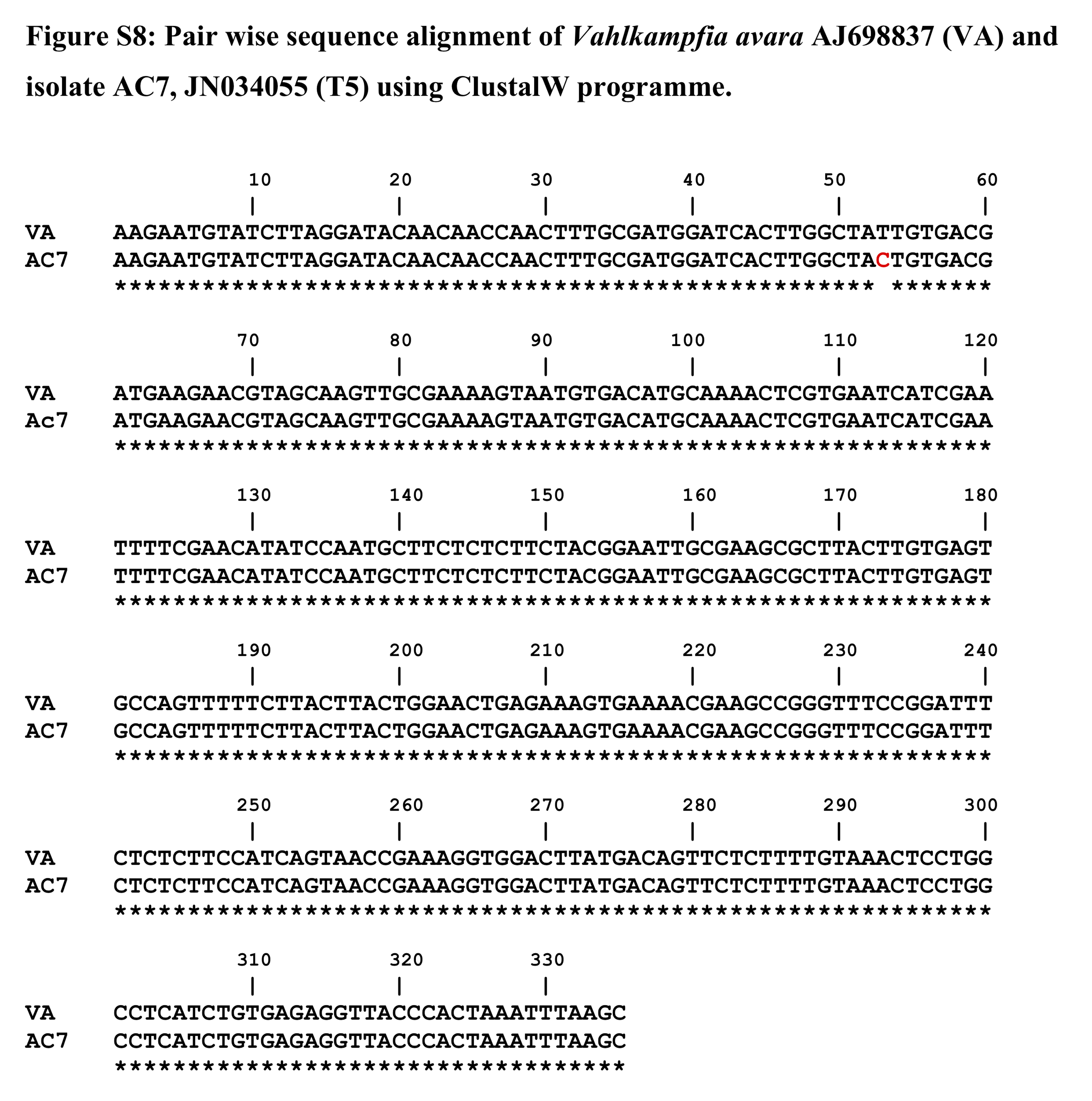

Supplement: Figure S8 — Pair wise sequence alignment of Vahlkampfia avara AJ698837 (VA) and isolate AC7, JN034055 (T5) using ClustalW programme. Homologous residue (*), non homologous residue (blank), base substitution or insertion (red colour font). (TIF) [file pone.0024327.s008.tif]

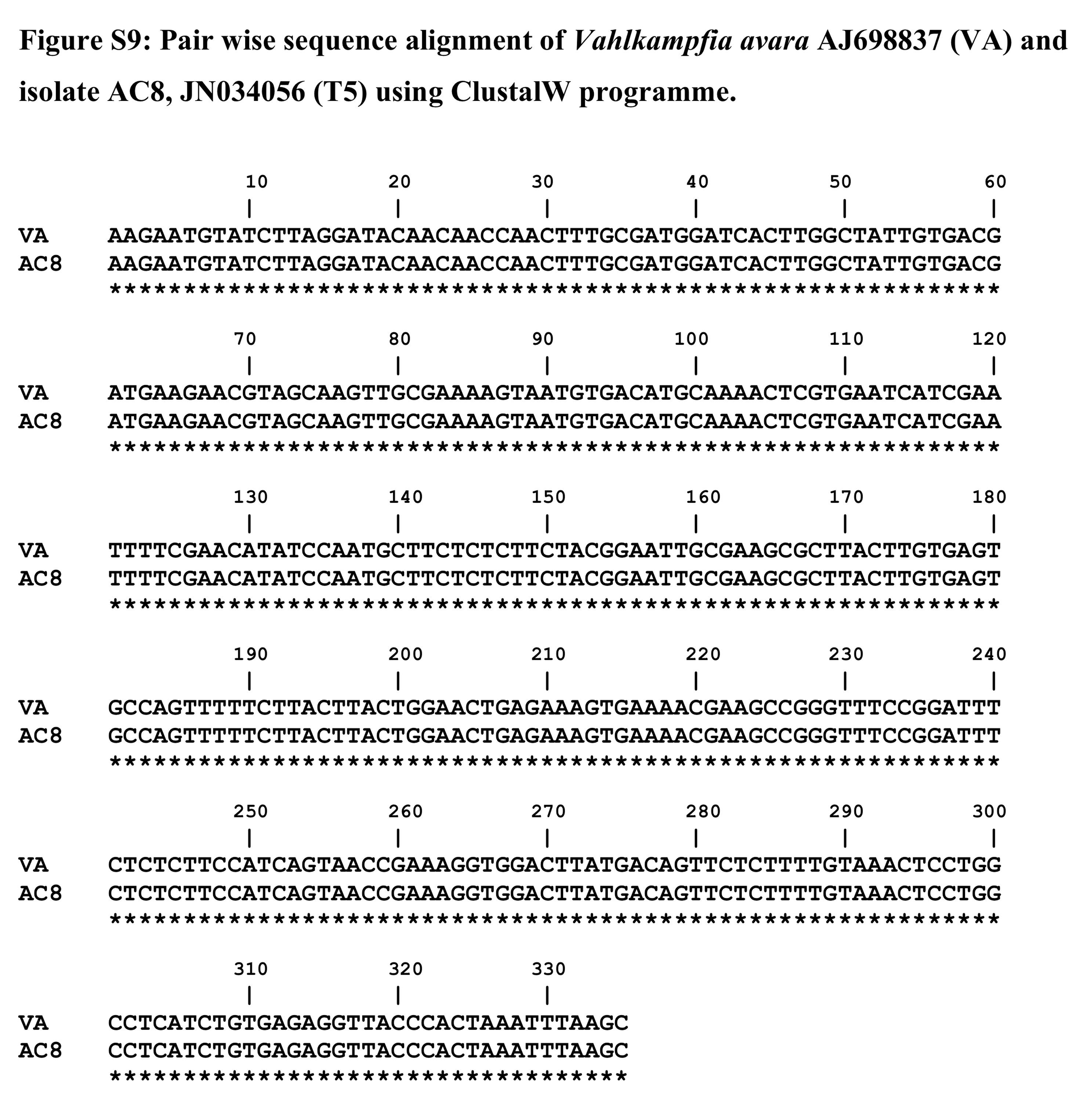

Supplement: Figure S9 — Pair wise sequence alignment of Vahlkampfia avara AJ698837 (VA) and isolate AC8, JN034056 (T5) using ClustalW programme. Homologous residue (*). (TIF) [file pone.0024327.s009.tif]
